# Usefulness of Respiratory Mechanics and Laboratory Parameter Trends as Markers of Early Treatment Success in Mechanically Ventilated Severe Coronavirus Disease: A Single-Center Pilot Study

**DOI:** 10.3390/jcm10112513

**Published:** 2021-06-06

**Authors:** Daisuke Kasugai, Masayuki Ozaki, Kazuki Nishida, Hiroaki Hiraiwa, Naruhiro Jingushi, Atsushi Numaguchi, Norihito Omote, Yuichiro Shindo, Yukari Goto

**Affiliations:** 1 Department of Emergency and Critical Care Medicine, Nagoya University Graduate School of Medicine, Tsurumai-cho 65, Syowa-ku, Nagoya, Aichi 466-8550, Japan; mozaki@med.nagoya-u.ac.jp (M.O.); hiraiwa.hiroaki@med.nagoya-u.ac.jp (H.H.); jinjin435@yahoo.co.jp (N.J.); nummer0116@gmail.com (A.N.); gotoyu@med.nagoya-u.ac.jp (Y.G.); 2 Department of Biostatistics Section, Center for Advanced Medicine and Clinical Research, Nagoya University Graduate School of Medicine, 65, Tsurumaicho, Showa, Nagoya, Aichi 466-8550, Japan; nishida@med.nagoya-u.ac.jp; 3 Department of Respiratory Medicine, Nagoya University Graduate School of Medicine, Tsurumai-cho 65, Syowa-ku, Nagoya, Aichi 466-8550, Japan; nori920@med.nagoya-u.ac.jp (N.O.); yshindo@med.nagoya-u.ac.jp (Y.S.)

**Keywords:** COVID-19, mechanical ventilation, respiratory failure

## Abstract

Whether a patient with severe coronavirus disease (COVID-19) will be successfully liberated from mechanical ventilation (MV) early is important in the COVID-19 pandemic. This study aimed to characterize the time course of parameters and outcomes of severe COVID-19 in relation to the timing of liberation from MV. This retrospective, single-center, observational study was performed using data from mechanically ventilated COVID-19 patients admitted to the ICU between 1 March 2020 and 15 December 2020. Early liberation from ventilation (EL group) was defined as successful extubation within 10 days of MV. The trends of respiratory mechanics and laboratory data were visualized and compared between the EL and prolonged MV (PMV) groups using smoothing spline and linear mixed effect models. Of 52 admitted patients, 31 mechanically ventilated COVID-19 patients were included (EL group, 20 (69%); PMV group, 11 (31%)). The patients’ median age was 71 years. While in-hospital mortality was low (6%), activities of daily living (ADL) at the time of hospital discharge were significantly impaired in the PMV group compared to the EL group (mean Barthel index (range): 30 (7.5–95) versus 2.5 (0–22.5), *p* = 0.048). The trends in respiratory compliance were different between patients in the EL and PMV groups. An increasing trend in the ventilatory ratio during MV until approximately 2 weeks was observed in both groups. The interaction between daily change and earlier liberation was significant in the trajectory of the thrombin–antithrombin complex, antithrombin 3, fibrinogen, C-reactive protein, lymphocyte, and positive end-expiratory pressure (PEEP) values. The indicator of physiological dead space increases during MV. The trajectory of markers of the hypercoagulation status, inflammation, and PEEP were significantly different depending on the timing of liberation from MV. These findings may provide insight into the pathophysiology of COVID-19 during treatment in the critical care setting.

## 1. Introduction

The number of patients with coronavirus disease (COVID-19) is increasing worldwide, including in Japan. In Japan, 8.1% of all COVID-19 cases require mechanical ventilation (MV), and the 30-day mortality rate has been reported to be 30% [1,2,3]. COVID-19 requires a longer treatment duration than other causes of viral pneumonia, with a median length of stay in the intensive care unit (ICU) of 10 days [4]. Once the capacity of ICU services for COVID-19 is overwhelmed, a significant increase in mortality and excess mortality from any cause may be expected [3,5]. Furthermore, prolonged MV is a risk factor for ICU-acquired weakness [6]. In this context, whether the patient with severe COVID-19 will be liberated from MV is of particular interest for improving patients’ outcomes.

Thus far, little is known about the time course of COVID-19-related respiratory failure during ICU treatment. Previous studies have suggested that severe COVID-19 is characterized by excessive inflammation and hypercoagulation [7,8,9]. In addition to the conventional acute respiratory distress syndrome phenotype, there is another phenotype of high pulmonary compliance and increased physiologic dead space, which is thought to be due to pulmonary microthrombosis [10]. Meanwhile, lower compliance was reported to be associated with prolonged MV, which is similar to findings in other causes of acute respiratory distress syndrome [11]. Considering the complexity of the pathophysiology of severe COVID-19, knowledge of how time series data of clinical parameter changes is needed to assess the response to treatment and to make clinical decisions. However, it is poorly documented how respiratory and laboratory findings—including respiratory compliance, physiologic dead space, and inflammatory and coagulation biomarkers of severe COVID-19—change in response to empirical treatment, including anti-viral medication usage, anti-coagulation, or corticosteroid administration.

The aim of this study was to characterize the time course of the parameters and outcomes of severe COVID-19 in relation to the timing of liberation from MV.

## 2. Materials and Methods

### 2.1. Ethics Statements

The Nagoya University Hospital Institutional Review Board approved this study (registration number: 2020-0519), and informed consent of the participants was waived but the opt-out method was adopted according to the ethics guidelines.

### 2.2. Study Design, Setting, and Population

To characterize the time course of the parameters and outcomes of severe COVID-19 in relation to the timing of liberation from MV, we conducted a retrospective observational study at Nagoya University Hospital from 1 March 2020 to 15 December 2020. Nagoya University Hospital is a quaternary academic medical center with 1035 beds, including 10 emergency and medical ICU (EMICU) beds and 30 surgical ICU beds, located in the Aichi Prefecture, one of the epicenters of COVID-19 from the first wave of the pandemic in Japan. The EMICU usually treats 10–20 patients with extracorporeal membrane oxygenation (ECMO) annually for the management of severe respiratory failure or cardiogenic shock. All severe COVID-19 cases in the hospital and transfers from other hospitals, which are coordinated by the Infectious Disease Control Office in Nagoya City, were admitted to the air-isolated beds of the EMICU. Patients requiring less than 4 L of oxygen were transferred to another COVID-19 ward.

Eligible patients in this study had COVID-19 that required MV. Exclusion criteria were patients introduced to venovenous (VV)-ECMO. The diagnosis of COVID-19 was confirmed by real-time polymerase chain reaction test of severe acute respiratory syndrome coronavirus 2 (SARS-CoV-2) from any specimen. Patients were categorized into the early liberation from ventilation group (EL group) or prolonged MV group (PMV group). Early liberation from MV was defined as successful extubation within 10 days of MV, since 10 days is the widely adopted duration of antiviral and steroid treatment [12,13].

### 2.3. Management of Coronavirus Disease

All mechanically ventilated patients with COVID-19 were initially managed with pressure-controlled ventilation. Placement in the prone position was considered when the PaO_2_/FiO_2_ ratio was less than 150, and was performed at the physicians’ discretion. Neuromuscular blockade was administered for less than 48 h when significant patient ventilator desynchrony was observed. All patients received favipiravir or remdesivir as antiviral medications, depending on clinical availability. A 10-day course of intravenous dexamethasone (6.6 mg) once daily was initially started [12]. Antibiotics were administered to patients with suspected bacterial co-infections. Unfractured heparin was administered and titrated to maintain the activated prothrombin time ratio between 1.5 and 2.5 after MV in all patients [14]. Tracheostomy was considered if patients could not be extubated within 10 days [15]. Because of inadequate personal protective equipment and concerns about nosocomial infections, physiotherapists were unable to be directly involved in bedside rehabilitation [16]. The bedside rehabilitation was performed by a physiotherapist after negative conversion of the SARS-CoV-2 PCR test result, or it was performed by doctors and nurses under the supervision of a physiotherapist after the patient was liberated from MV.

### 2.4. Data Collection

Demographic information was extracted from patients’ electronic medical records. The details of the parameters during ICU management were extracted from the ICU patient information system (Fortec ACSYS, Phillips Japan). Ventilator parameters were recorded minutely by the IntelliVue MX800 (Philips Japan). Static compliance was calculated using the tidal volume and driving pressure. As an indicator of physiologic dead space, the ventilatory ratio was calculated using the following formula: [minute ventilation (mL/min) ×  partial pressure of carbon dioxide (mm Hg)]/(predicted body weight × 100 × 37.5) [17,18]. The following laboratory parameters were routinely monitored daily during MV and extracted from the database: coagulation markers (D-dimer, thrombin-antithrombin complex (TAT), plasmin-alpha2-plasmininhibitor-complex, fibrin degradation products (FDP), antithrombin 3 (AT3), fibrinogen, activated partial thromboplastin time ratio, and platelet count), biomarkers of inflammation and lung injury (C-reactive protein level, procalcitonin (PCT) level, ferritin level, white blood cell count, neutrophil count, lymphocyte count, 50% hemolytic complement activity (CH50), and Krebs von den Lungen-6 (KL-6)). Activities of daily living (ADL) before admission and at the time of hospital discharge were measured using the Barthel index, which was routinely evaluated by the nurses and recorded in the nursing summary [19,20].

### 2.5. Statistical Analysis

Continuous data are summarized as median and interquartile range (25th–75th percentiles). Categorical variables are expressed as numbers (%). Non-parametric variables were compared between the EL and PMV groups using the Mann–Whitney U test. The Barthel index at hospital discharge was compared between the groups, and the median Barthel index of each component in both groups was visualized using a Rader chart. Nonparametric trending changes in each parameter in both groups were fitted by smoothing splines. Additionally, multivariable mixed effect linear regression models were used to evaluate the longitudinal associations between daily changes in each parameter during initial 5 days and the EL group [21]. Variables were excluded from this evaluation when the linearity assumption seems to be inappropriate, by judging from the spline regression analysis. Within-subject changes were included in the model as random effects to adjust for patient factors. Early liberation, days after MV, and their interaction were assumed as fixed effects in the model. When the interaction term was statistically significant, we considered that the trajectory of the parameter was different between the two groups. Using the parameters that showed significant differences in daily changes that interacted with early liberation in the linear mixed effect model, the trajectory of each parameter was converted into the coefficient using linear regression model, and finally converted into the EL prediction score. The cutoff of each coefficient was determined by the results of the linear mixed effect model. Receiver operating characteristic (ROC) curve analysis was subsequently used to evaluate the performance of the predictive score. For missing data, the number of missing values were reported and complete-case analysis was performed. All analyses were performed using R software (version 4.0.2; The R Foundation).

## 3. Results

### 3.1. Patient Characteristics and Outcomes

**Figure 1:**
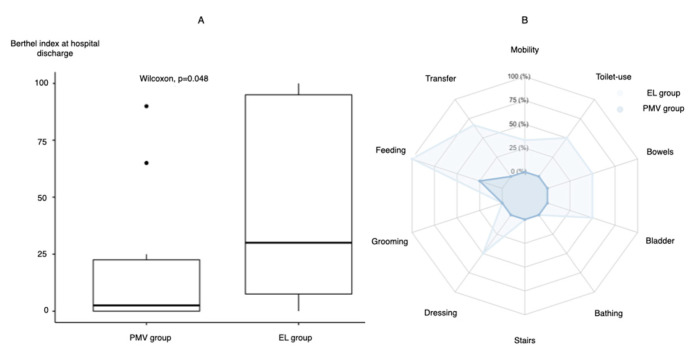
Comparison of the Barthel index at hospital discharge between the early liberation and prolonged mechanical ventilation groups. (**A**) Comparison between the two groups. (**B**) Radar chart of each component of the Barthel index. EL group, early liberation from ventilation group; PMV group, prolonged mechanical ventilation group.

Of the 52 patients with COVID-19 admitted to the EMICU during the study period, 31 of 32 mechanically ventilated patients were included in this study; one patient required VV-ECMO and was excluded (Supplementary Figure S1: additional file S1). The details of the baseline characteristics are shown in Table 1. The median age of the patients was 71 years. Most patients did not require nursing care before admission (median Barthel index: 100) and were more likely to be male (86%). Common comorbidities included diabetes mellitus (58%) and hypertension (38%). Twenty cases were successful in early liberation from MV (69%). The median worst partial pressure of oxygen/fraction of inspired oxygen (P/F) ratio was found to be 96. The initial ventilatory parameters did not differ between the groups. D-dimer levels were slightly elevated in the PMV group compared to the MV group. Overall, in-hospital mortality was low (6%), and one patient developed massive ischemic stroke after extubation and was withdrawn from care. Figure 1A,B shows the Barthel indexes of both groups at hospital discharge. ADL at the time of hospital discharge was significantly impaired in the PMV group compared to the EL group (median Barthel index (range): 30 (7.5–95) versus 2.5 (0–22.5), *p* = 0.048).

### 3.2. Ventilatory and Laboratory Parameters and Liberation from Mechanical Ventilation

**Figure 2:**
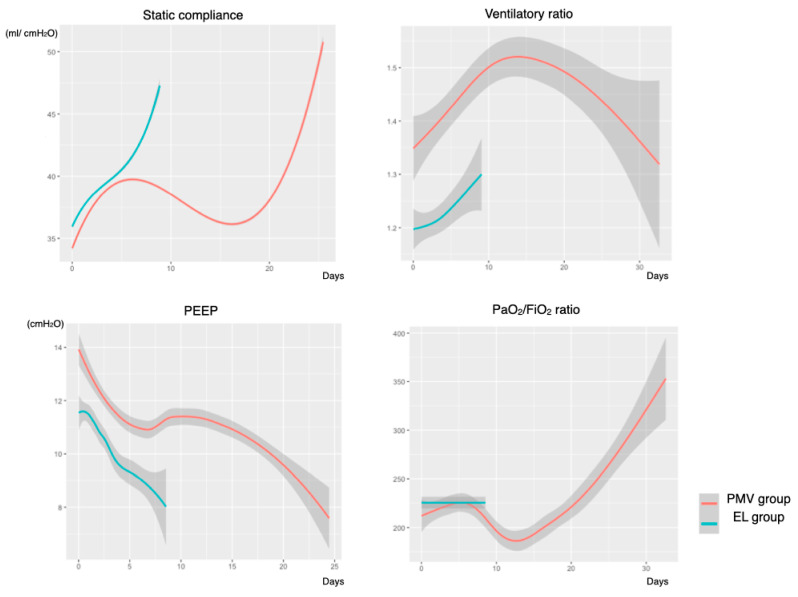
Trend of respiratory mechanic parameters in relation to the timing of liberation from mechanical ventilation. PEEP, positive end-expiratory pressure; EL group, early liberation from ventilation group; PMV group, prolonged mechanical ventilation group. The number of study timepoint: static compliance, 474,429; ventilatory ratio, 1813; PEEP, 474,941; PaO_2_/FiO_2_ ratio, 1778.

Figure 2 shows the trends in ventilatory parameters in each group. The EL group was managed with a lower PEEP throughout the period. Trends of compliance and the P/F ratio were different between the EL and PMV groups with an inflection point on day 5 of MV. The ventilatory ratio was higher in the PMV group than in the EL group. Of note, an increasing trend in the ventilatory ratio during MV until approximately 2 weeks was observed in both groups.

**Figure 3:**
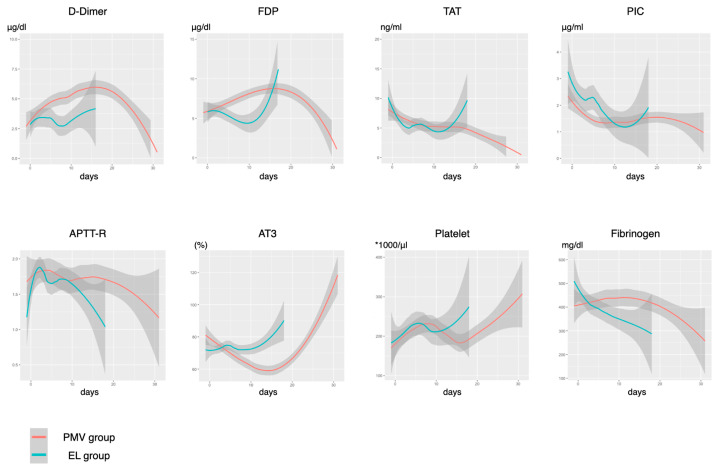
Trends of the coagulation parameters. FDP, fibrin degradation products; TAT, thrombin-antithrombin complex; PIC, plasmin-alpha2-plasmininhibitor-complex; APTT-R, activated partial thromboplastin time ratio; AT3, antithrombin 3; EL group, early liberation from ventilation group; PMV group, prolonged mechanical ventilation group. The number of study timepoint: D-dimer, 393; FDP, 392; TAT, 355; PIC, 354; APTT-R, 394; AT3, 392; platelet, 394; FG, 394.

**Figure 4:**
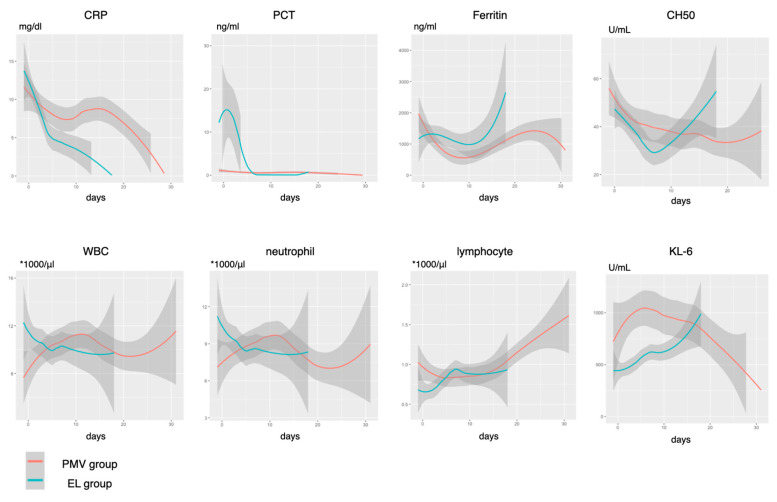
Trends of the laboratory parameters of inflammation. CH50, 50% hemolytic complement activity; CRP, C-reactive protein; PCT, procalcitonin; WBC, white blood cell; KL6, Krebs von den Lungen-6; EL group, early liberation from ventilation group; PMV group, prolonged mechanical ventilation group. The number of study timepoint: CRP, 394; PCT, 392; Ferritin, 349; CH50, 198; WBC, 396; neutrophil, 393; lymphocyte, 393; KL6, 370.

Figure 3 and Figure 4 show each trend of laboratory parameters according to the duration of MV. Despite appropriate therapeutic anticoagulation, D-dimer and FDP levels were gradually increased and the AT3 level was decreased until day 14 in the PMV group. A decrease in the platelet count was not observed. In terms of inflammatory biomarkers, CRP levels were continuously high in the PMV group. PCT levels were initially high in patients with successful early liberation, and then they immediately became negative. The ferritin levels increased in both groups at about 2 weeks, but a significant difference in their trajectory was not observed. While the CH50 level was decreased within the normal range, it increased in the EL group. KL-6 levels were significantly high initially in the PMV group, but elevation of KL-6 levels was observed in both groups.

The results of the longitudinal association between daily changes in each parameter during the initial 5 days and early liberation from MV are shown in Supplementary Figure S2 (Additional file S2). We found that CRP (*p* = 0.048), TAT (*p* = 0.019), fibrinogen (*p* = 0.002), AT3 (*p* < 0.001), lymphocyte (*p* = 0.009), and PEEP (*p* < 0.001) values showed significantly different daily changes that interacted with early liberation. An EL prediction score was developed using the trajectory of these variables (Supplementary Figure S3: Additional file S3). The area under ROC for the prediction of early liberation (95 %CI) was 0.913 (0.823–1), which was significantly higher than other severity scales (0.573 (0.34–0.802), 0.47 (0.262–0.679), and 0.457 (0.225–0.689) for the APACHEII, SOFA, and 4C mortality scores, respectively).

## 4. Discussion

The main findings of this study are as follows: (1) prolonged MV was significantly associated with poor ADL at discharge in the setting of rehabilitation-limiting situations; (2) the trajectory of ventilator and laboratory data were characterized between patients with early liberation and prolonged MV; and (3) early-phase differences in the trajectories of hypercoagulability, inflammatory, and PEEP markers were observed depending on the timing of liberation from MV, which can potentially be useful in identifying patients with early treatment success.

In this single-center observational study, the mortality rate was low compared to that in previous reports [1,2,3]. However, patients with prolonged MV showed significantly poor ADL. The relationship between the length of MV and ADL is well-known [22]. The ADL impairment may be due to clinical setting characteristics in the management of severe COVID-19, i.e., bedside interventions of rehabilitation were significantly impaired in our hospital because of physiotherapists’ concerns of exhaustion of personal protective equipment and nosocomial infection. Although we could not evaluate the long-term outcome of quality of life, our findings indicate that post-intensive care syndrome is particularly important in COVID-19 patients with prolonged MV. This finding may aid in future clinical decision-making and policymaking in terms of staffing and resource allocation in critical care settings during ongoing pandemics. Our findings indicate the importance of direct intervention by physiotherapists in the management of COVID-19. The duration of MV may be used as a surrogate marker for ADL impairment after treatment.

We observed a decreasing trend in respiratory static compliance despite the higher PEEP setting after day 5 and a higher ventilatory ratio in patients with prolonged MV than in those with early liberation. In patients with worsening COVID-19, two types of pathophysiologies may explain this change: pulmonary micro-thromboembolism and organizing pneumonia [23]. Our findings were consistent with those of previous reports in that therapeutic anticoagulation was not fully controlled coagulopathy in COVID-19 [24]. The decrease in the AT3 level and continuous elevation of the TAT suggests poorly controlled thrombin activity during the treatment. The role of complement activation in thrombotic tendency uncontrolled by heparin has been previously documented [25]. In this study, complements were gradually consumed during the treatment phase, which is consistent with previous findings [26]. Meanwhile, the combination of the elevation of the KL-6 level, a marker of interstitial lung injury, worsening respiratory compliance with poor recruitment, and increased physiologic dead space may be explained by the ongoing fibrin deposition of organizing pneumonia [27,28]. This is consistent with the pathologic findings of acute fibrinous and organizing pneumonia-predominant histology in the later phase of treatment, and this may explain the downward trend in compliance despite the higher PEEP setting [29,30]. To further understand the underlying mechanism in the exacerbating conditions, prospective studies with computed tomography pulmonary angiography and/or bronchoalveolar lavage evaluation may be warranted.

Notably, an increasing trend in the ventilatory ratio was also observed in patients with early liberation and in patients with prolonged ventilation. Taken together, these findings may indicate that empirical therapeutic anticoagulation and a 10-day course of dexamethasone (6.6 mg) were not enough to manage the underlying mechanisms. Recently, the CoDEX trial showed that administration of a higher dose of dexamethasone for severe COVID-19 shortened the duration of MV [31]. Further evaluation of anticoagulation and more intensive anti-inflammatory management may be warranted in patients with prolonged ventilation. The trajectory of respiratory compliance and oxygenation was different between the two groups after day 5 of MV. Early tracheostomy was associated with early liberation from MV and preserved ADL [32,33]. It may be reasonable to make a clinical decision for additional treatment or earlier tracheostomy by reviewing the clinical time course until about day 5 of MV.

This study has several limitations. Firstly, because of the nature of the single-center observational study with a small sample size, we mainly focused on descriptive analysis. Furthermore, selection bias might have occurred in inter-hospital transfers, which may limit the external validity of our study. The prognostic value of each parameter and the predictive score should be evaluated in further multicenter studies. Secondly, the titration of PEEP was not protocolized and carried out according to bedside clinicians’ preferences. Although a higher PEEP setting was used in patients with prolonged MV, it is unclear whether these patients require a higher PEEP setting because of poor oxygenation or if an unnecessarily high PEEP setting was prescribed, as this may worsen the ventilation perfusion mismatch [34,35,36].

## 5. Conclusions

Prolonged MV was associated with poor ADL at hospital discharge during COVID-19 infection. The indicator of physiological dead space increases during MV. The trajectory of markers of the hypercoagulation status, inflammation, and PEEP were significantly different depending on the timing of liberation from MV. These findings may provide insight into the pathophysiology of COVID-19 during treatment in a critical care setting.

## Figures and Tables

**Table 1:** Patient characteristics.

Characteristic	Total Patients (31)	Early Liberation from MV		*p* Value
		Success (20)	Failure (11)	
Age, years, median (IQR)	71 (64–76)	71 (64–77)	70 (56–73)	0.535
Male sex, n (%)	25 (81)	17 (85)	8(73)	0.546
BMI (kg/m^2^), median (IQR)	24.5 (21.8–28.5)	25.2 (21.5–28.5)	23.2 (22.4– 30.2)	0.67
Comorbidities, n, median (IQR)	1 (1–2)	1 (1–2)	2 (1–2)	0.118
HT, n (%)	11 (35)	6 (30)	5 (45)	0.452
DM, n (%)	18 (58)	10 (50)	8 (73)	0.275
Chronic heart failure, n (%)	3 (9.6)	1 (5)	2 (18)	0.281
End-stage renal disease, n (%)	5 (16)	2 (10)	3 (27)	0.317
Cancer, n (%)	2 (6.4)	1 (5)	1 (9.1)	>0.99
Chronic pulmonary disease, n (%)	2 (6.4)	1 (5)	1 (9.1)	>0.99
Dementia, n (%)	3 (9.6)	3 (15)	0 (0)	0.535
4C mortality score, median (IQR)	12 (11–14)	12 (11–13)	12 (11–14)	0.707
SOFA score, median (IQR)	7 (6–10)	7 (5–10)	7 (6–10)	0.802
APACHE II score, median (IQR)	13 (11–19)	13 (11–19)	15 (11–19)	0.521
Parameters at the time of MV				
PaO_2_/FiO_2_ ratio	96 (82–114)	85 (81–114)	101 (89–113)	0.47
Static compliance, mL/cmH_2_O, median (IQR)	38 (33–42)	38 (34–43)	38 (33–39)	0.614
Ventilatory ratio, median (IQR)	1.26 (1.17–1.41)	1.24 (1.17–1.32)	1.38 (1.18–1.58)	0.119
PEEP, cmH_2_O	11 (10–14)	10 (10–12)	14 (10–15)	
NMB, n (%)	5 (16)	4 (20)	1 (9.1)	
Prone positioning, n (%)	13 (42)	7 (35)	6 (55)	0.477
D-dimer level, μg/mL, median (IQR)	2.31 (1.52–4.54)	1.63 (1.09–4.18)	3.36 (2.35–10.1)	0.132
Treatment				
Anti-viral, n (%)				
Favipiravir	15 (48)	8 (40)	7 (64)	
Remdesivir	16 (52)	13 (65)	3 (27)	
Steroid, n (%)	26 (84)	19 (95)	7 (64)	0.0416
Initial antibacterial drug, n (%)	21 (68)	14 (70)	7 (64)	>0.99
Tracheostomy, n (%)	7 (23)	0 (0)	7 (64)	<0.001
Duration of mechanical ventilation (days), median (IQR)	10 (5–20)	6 (4–9)	24 (20–30)	<0.001
ICU stay, median (IQR)	12 (10–20)	11 (8–12)	27 (21–36)	<0.001
In-hospital mortality, n (%)	2 (6.5)	1 (5)	1 (9.1)	>0.99
Barthel index at discharge, median (IQR)	20 (0–65)	30 (7.5–95)	2.5 (0–22.5)	0.048

BMI, body mass index; HT, hypertension; DM, diabetes mellitus; SOFA, Sequential Organ Failure Assessment; APACHE II, acute physiology and chronic health evaluation II; MV, mechanical ventilation; PaO_2_/FiO_2_, partial pressure of oxygen/fraction of inspired oxygen; PEEP, positive end-expiratory pressure; NMB, neuromuscular blockade; ICU, intensive care unit.

## Data Availability

The dataset supporting the conclusions of this article is available from the corresponding author on reasonable request.

## Supplementary Materials

The following are available online at https://www.mdpi.com/article/10.3390/jcm10112513/s1, Supplementary Figure S1: Flow diagram of patient selection, Supplementary Figure S2: Association between daily change in each parameter during the initial 5 days and early liberation from mechanical ventilation. CRP, C-reactive protein; FDP, fibrin degradation products; TAT, thrombin-antithrombin complex; PIC, plasmin-alpha2-plasmininhibitor-complex; AT3, antirhombin3; KL6, Krebs von den lungen-6; CH50, 50% hemolytic complement activity; EL group, early liberation from ventilation group; PMV group, prolonged mechanical ventilation group. The number of missing values were following: KL6, 13; _TAT, 16; PIC, 16; ferritin, 16; CH50, 63, Supplementary Figure S3: The components of the EL prediction score and its performance. The cutoff coefficient of each component was determined by the estimated effect of daily change and its interaction with early liberation (A). The area under the receiver operating characteristic curve of the EL prediction score was significantly high compared to other severity scales (B). PEEP, positive end-expiratory pressure; TAT, thrombin-antithrombin complex; AT3, antirhombin3; CRP, C-reactive protein; EL, early liberation; APACHE II, acute physiology and chronic health evaluation II; SOFA, Sequential Organ Failure Assessment.

## Abbreviations

COVID-19, coronavirus disease; MV, mechanical ventilation; ICU, intensive care unit; EMICU, emergency and medical intensive care unit; ECMO, extracorporeal membrane oxygenation; VV, venovenous; SARS-CoV-2, severe acute respiratory syndrome coronavirus 2; EL group, early liberation from ventilation group; PMV group, prolonged mechanical ventilation group; CH50, 50% hemolytic complement activity; ADL, activities of daily living; P/F, partial pressure of oxygen/fraction of inspired oxygen; TAT, thrombin-antithrombin complex; FDP, fibrin degradation products; AT3, antithrombin 3; PCT, procalcitonin; KL-6, Krebs von den Lungen-6.
