# Supplementary figures and images for: Usefulness of Respiratory Mechanics and Laboratory Parameter Trends as Markers of Early Treatment Success in Mechanically Ventilated Severe Coronavirus Disease: A Single-Center Pilot Study
Source: J Clin Med. 2021 Jun 6;10(11):2513. doi: 10.3390/jcm10112513 (PMC8201161; doi:10.3390/jcm10112513)

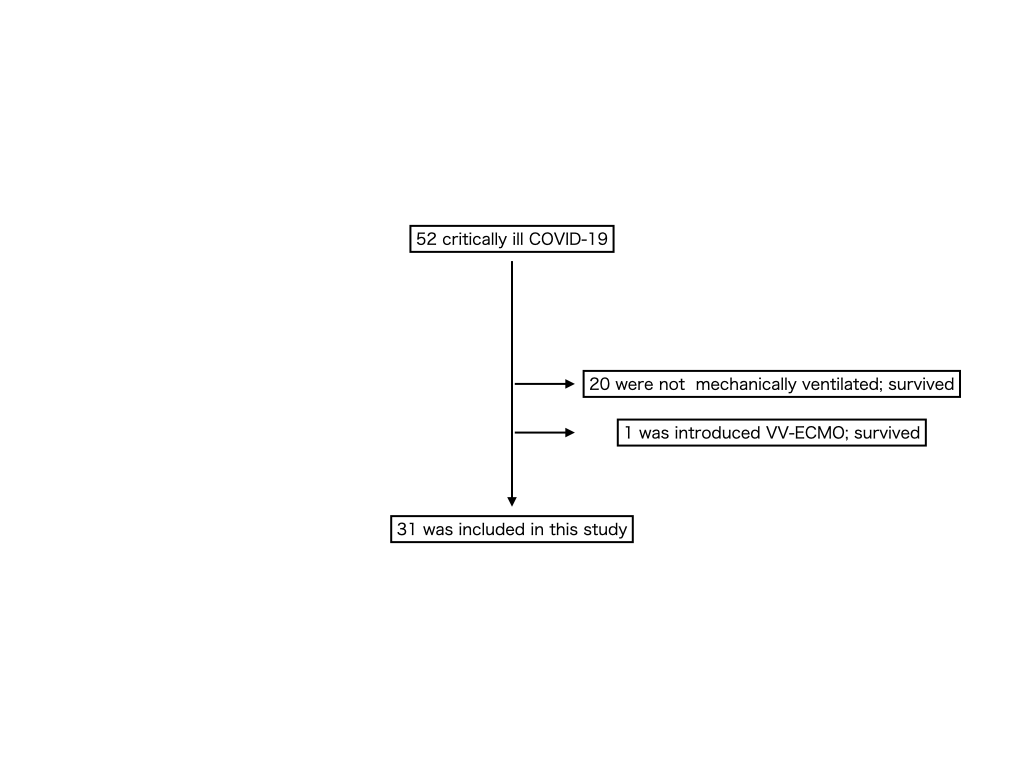

Supplement: Supplementary file 1 [file jcm-10-02513-s001.zip › supplemental_figures/supplement_figure1.tiff]

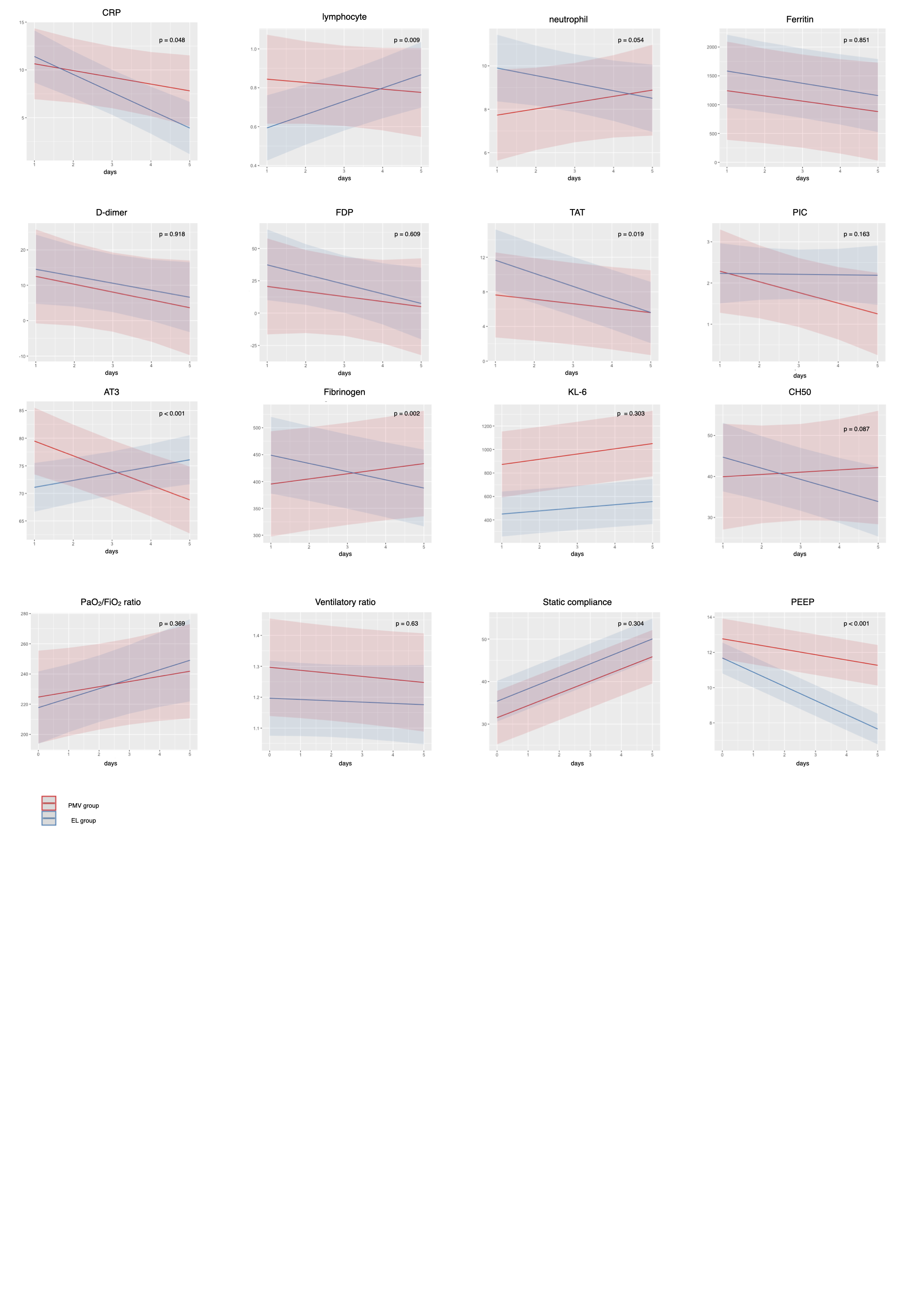

Supplement: Supplementary file 1 [file jcm-10-02513-s001.zip › supplemental_figures/supplemental_figure_2.tiff]

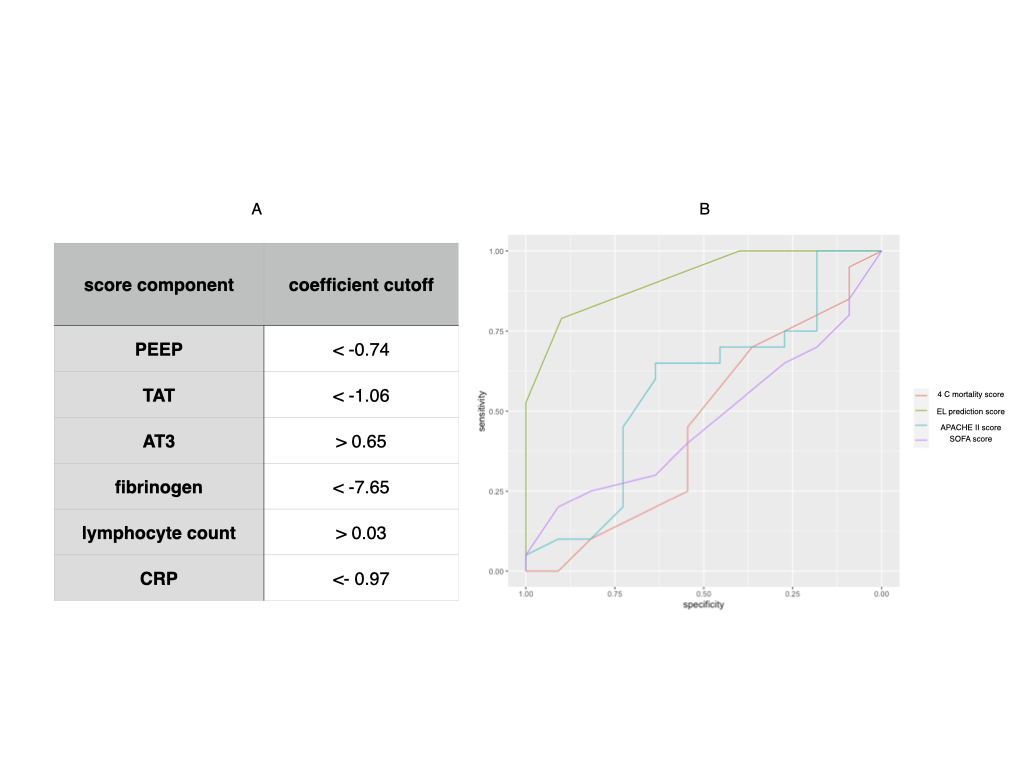

Supplement: Supplementary file 1 [file jcm-10-02513-s001.zip › supplemental_figures/supplement_figure3.tiff]
